# Longer-term outcome in the prevention of psychotic disorders by the Vienna omega-3 study

**DOI:** 10.1038/ncomms8934

**Published:** 2015-08-11

**Authors:** G. Paul Amminger, Miriam R. Schäfer, Monika Schlögelhofer, Claudia M. Klier, Patrick D. McGorry

**Affiliations:** 1 Orygen—The National Centre of Excellence in Youth Mental Health, The University of Melbourne, 35 Poplar Road, Parkville, Victoria 3052, Australia; 2 Department of Child and Adolescent Psychiatry, Medical University of Vienna, Vienna 1090, Austria; 3 Department of Pediatrics and Adolescent Medicine, Medical University of Vienna, Vienna 1090, Austria

## Abstract

Long-chain omega-3 polyunsaturated fatty acids (PUFAs) are essential for neural development and function. As key components of brain tissue, omega-3 PUFAs play critical roles in brain development and function, and a lack of these fatty acids has been implicated in a number of mental health conditions over the lifespan, including schizophrenia. We have previously shown that a 12-week intervention with omega-3 PUFAs reduced the risk of progression to psychotic disorder in young people with subthreshold psychotic states for a 12-month period compared with placebo. We have now completed a longer-term follow-up of this randomized, double-blind, placebo-controlled trial, at a median of 6.7 years. Here we show that brief intervention with omega-3 PUFAs reduced both the risk of progression to psychotic disorder and psychiatric morbidity in general in this study. The majority of the individuals from the omega-3 group did not show severe functional impairment and no longer experienced attenuated psychotic symptoms at follow-up.

## Abstract (web summary)

Long-chain omega-3 polyunsaturated fatty acids are essential for neural development and lack of these fatty acids has been implicated in a number of mental health conditions. Here the authors report the longer-term efficacy of a randomized, double-blind, placebo-controlled clinical trial assessing the effects of fish oil capsules in individuals at ultrahigh risk for psychosis.

## Graphical abstract

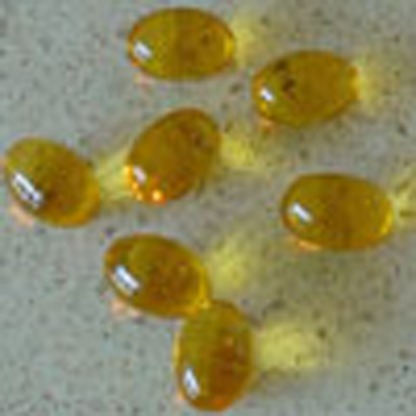

Schizophrenia is a devastating condition characterized by delusions, hallucinations and cognitive problems that typically manifests in adolescence or early adulthood. The onset may be abrupt or insidious, but the majority of individuals experience the slow and gradual development of a variety of clinically significant signs and symptoms. Schizophrenia does not just affect mental health; people with schizophrenia die more than a decade earlier than the general population, with this excess mortality largely due to cardiometabolic conditions^1^. Although early treatment has been linked to better outcomes^2^, current treatments for schizophrenia provide control rather than cure^3^.

For the past 20 years, there has been increasing academic and clinical interest in detecting and intervening in people presenting with potentially prodromal symptoms of psychosis^4^. This clinical syndrome has been termed an ‘at-risk mental state', and operationalized ‘ultrahigh risk' criteria have been developed to identify young people with this syndrome, which is associated with a very high risk of developing psychosis^5^,^6^. A recent meta-analysis in 2,502 at-risk individuals found that the cumulative rate of transition to psychosis increased over time, with 18%, 22%, 29% and 36% developing a psychotic disorder by 6 months, 1, 2 and 3 years, respectively^7^. These values are consistent with evidence that some at-risk patients develop psychosis after the first 24 months following presentation, when the risk of transition is thought to be maximal^8^.

Reductions in the cell membrane levels of the omega-3 and omega-6 polyunsaturated fatty acid (PUFA) series have been observed in patients with schizophrenia^9^. In addition, several controlled trials have shown that supplementation with omega-3 PUFAs can reduce psychotic symptoms^10^. Since omega-3 PUFAs have no clinically relevant adverse effects and are considered generally beneficial to health, they are ideal for indicated prevention of psychosis. Almost a decade ago, we successfully conducted the first randomized, double-blind, placebo-controlled trial showing that omega-3 PUFAs prevented a first episode of psychotic disorder for up to 1 year after baseline^11^. Here we report the longer-term efficacy of a 12-week intervention with fish oil capsules (providing omega-3 PUFAs, that is, 700 mg of eicosapentaenoic acid and 480 mg of docosahexaenoic acid daily) versus placebo capsules (matched in appearance and flavour with the active treatment) in individuals at ultrahigh risk for psychosis. We show that omega-3 PUFAs significantly reduced the risk of progression to psychotic disorder during the entire follow-up period. The overall psychiatric morbidity during the follow-up period was also significantly lower in the omega-3 PUFA group.

## Results

### Study sample

**Figure 1:**
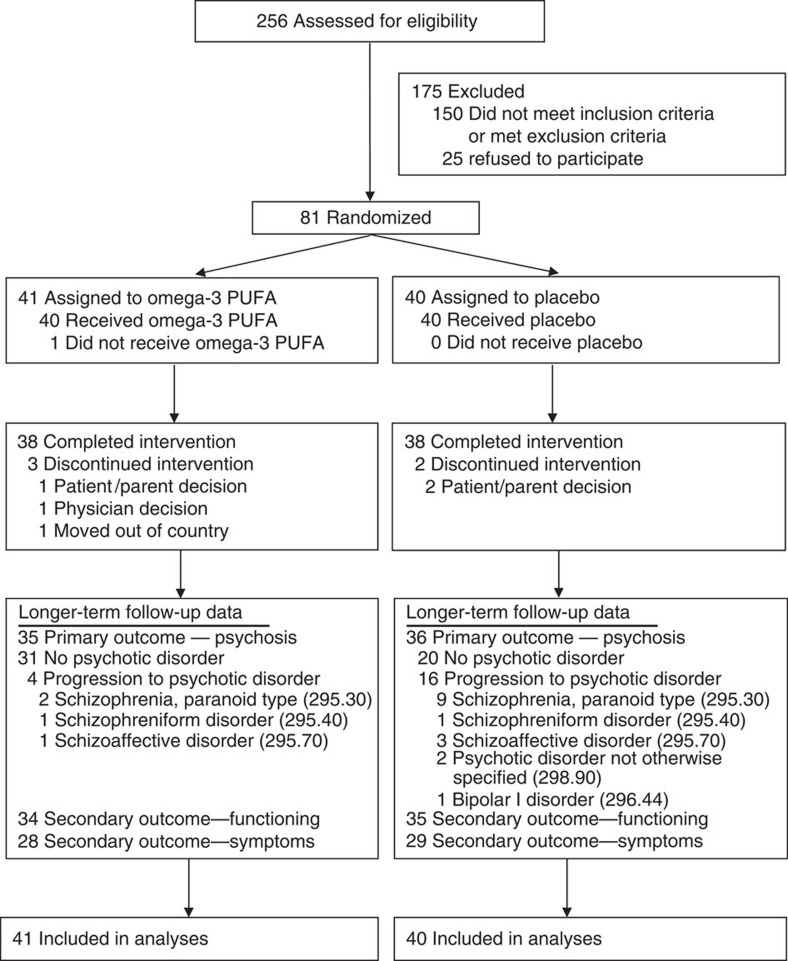
Enrolment and outcomes flowchart. We assessed 256 individuals for eligibility. Of those, 150 were excluded because they did not meet inclusion criteria or met exclusion criteria, while 25 refused participation. Eighty-one treatment-seeking individuals were enrolled in the trial, of which 41 were randomly assigned to omega-3 PUFAs and 40 to placebo. In each group, 38 individuals completed the intervention. Longer-term follow-up data on the primary outcome (that is, progression to psychotic disorder) were collected in 35 individuals from the omega-3 PUFA group and 36 individuals from the placebo group. Secondary outcome data on psychosocial functioning were collected in 34 and 35 individuals from the omega-3 and the placebo groups, respectively. Secondary outcome data on psychiatric symptoms, including positive symptoms, negative symptoms, general symptoms and depressive symptoms, were collected in 28 and 29 individuals from the omega-3 and the placebo group, respectively. All individuals enrolled in the trial were included in the data analysis.

Participants were aged 13–25 years at first presentation and met criteria for one or more of the three operationally defined and well-validated groups of risk factors for psychosis proposed by Yung *et al*.^12^: attenuated positive psychotic symptoms; transient psychosis; and genetic risk plus a decrease in functioning. Eighty-one treatment-seeking individuals were enrolled in the study, with 41 assigned to the omega-3 PUFA group and 40 to the placebo group; all patients were included in the intention-to-treat analysis (Fig. 1). The intake criteria the individuals met are as follows: attenuated psychotic symptoms (group 1; 49.4%, 40/81); transient psychosis (group 2; 7.4%, 6/81); trait plus state risk factors (group 3; 2.5%, 2/81); attenuated psychotic symptoms plus transient psychosis (groups 1 and 2; 35.8%, 29/81); and attenuated psychotic symptoms plus trait plus state risk factors (groups 1 and 3; 4.9%, 4/81). Fifty-four (67%, 54/81) patients were female, and the mean age (s.d.) at baseline was 16.4 (±2.1) years. Both treatment arms were comparable with respect to baseline characteristics, which included age, sex, body mass index, study entry criteria, illicit drug use, psychiatric symptoms and functioning, and erythrocyte fatty acid levels^11^. After randomization, participants received weekly assessments for 4 weeks, and then at 8 and 12 weeks (end of intervention), and subsequent follow-up at 6, 12 months and 7 years after baseline. The Positive and Negative Syndrome Scale (PANSS)^13^ and the Montgomery–Asberg Depression Rating Scale (MADRS)^14^ were used to examine psychiatric symptoms. The Global Assessment of Functioning (GAF) score was used as measure of functioning^15^. The Structured Clinical Interview for DSM-IV-TR Axis I Disorders (SCID-I/P)^16^ was used to ascertain psychiatric diagnoses. Detailed information on inclusion/exclusion criteria, randomization, blinding, study measures and inter-rater reliability is provided in the Methods section and elsewhere^11^.

### Conversion to psychotic disorder

The primary efficacy end point in the trial was conversion to psychotic disorder, which was operationally defined based on criteria by Yung *et al*.^12^, using severity thresholds on the PANSS^13^ proposed by Morrison and colleagues^17^. These levels had to be sustained for at least 1 week. The exit criteria marked the threshold (linked to positive psychotic symptoms) at which treatment with antipsychotic medication is usually initiated^18^. Seventy-one (87.7%, 71/81) individuals were successfully followed up ∼7 years after baseline. In 10 (12.3%, 10/81) subjects, information from their last follow-up assessment, which in all cases was the 12-month assessment, was used for the survival analysis. The median duration of follow-up in the sample was 6.7 years. The source of information to determine the longer-term outcome was interviews (63 with participants, 4 with next of kin) for 82.7% (67/81) of participants and hospital records for 4.9% (4/81). Of those with no longer-term data, six were from the omega-3 PUFA group and four from the placebo group. None of them has received psychiatric treatment according to a Vienna-wide electronic register of health service utilization since their initial presentation. According to the National Death Index, no participant had died.

**Figure 2:**
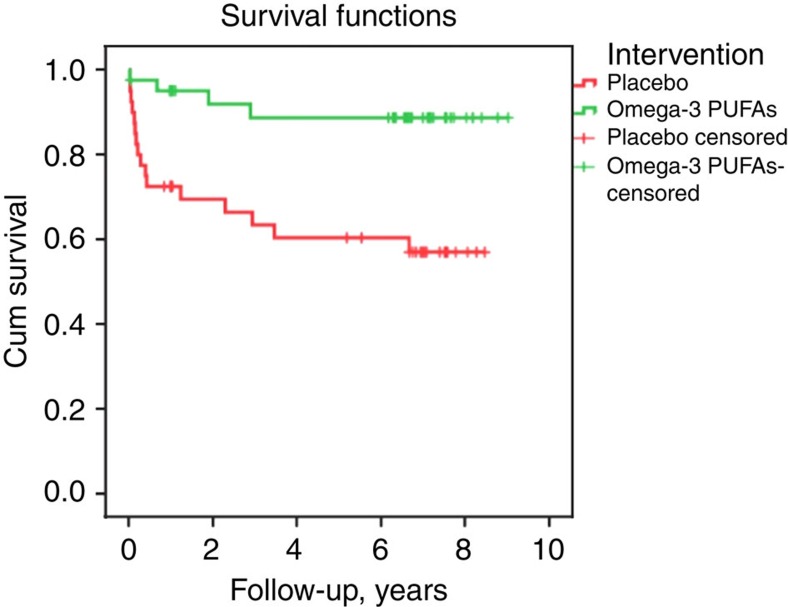
Kaplan–Meier estimates of the risk of progression from the at-risk state to psychotic disorder in participants assigned to omega-3 PUFAs or placebo. Four of 41 individuals from the omega-3 PUFA group and 16 of 40 individuals from the placebo group developed a psychotic disorder during the entire follow-up period. The difference between the groups in the cumulative risk of progression to psychosis was 30.2% (95% confidence interval, 10.1–50.4, with continuity correction). Kaplan–Meier survival analysis indicated the survival times were significantly different between the treatment groups, with a more rapid conversion time for the placebo group compared to the omega-3 PUFA group. *χ*^2^=9.84, *P*=0.002, log-rank test.

The cumulative conversion rate to psychosis at the longer-term follow-up was 9.8% (4/41) of subjects in the omega-3 PUFA group, and 40% (16/40) of subjects in the placebo group. The difference between the groups in the cumulative risk of progression to psychosis was 30.2% (95% confidence interval, 10.1–50.4, with continuity correction). Figure 1 shows the SCID-based DSM-IV lifetime diagnoses of the psychotic patients at longer-term follow-up. The survival times were significantly different between the treatment groups, with a more rapid conversion time for the placebo group compared with the omega-3 PUFA group (log-rank test: *χ*^2^=9.84, *P*=0.002) (Fig. 2). A sensitivity analysis assuming all cases that had no longer-term follow-up information would have developed psychosis was consistent with the intention-to-treat analysis (log-rank test, *χ*^2^=5.18, *P*=0.02).

### Secondary outcomes

Psychosocial functioning is another important outcome, independent of psychosis conversion^19^. In 69 individuals (85.2%, 69/81), a GAF score could be determined as a measure of functioning when exiting the trial (that is, at the last follow-up or at transition to a psychotic disorder) (Fig. 1). Repeated-measures mixed model analysis indicated a significant interaction between medication group and occasion for GAF scores (F_4,122.5_=2.67, *P*=0.035), and the omega-3 PUFA group had significantly higher functioning than the placebo group at longer-term follow-up (*P*=0.011). For the PANSS measures, interactions between medication group and occasion were significant for positive (F_4,133.6_=4.48, *P*=0.002), general (F_4,125.3_=4.52, *P*=0.002) and total scores (F_4,125.0_=4.59, *P*=0.002), and were trending towards significance for the negative scores (F_4,129.4_=2.16, *P*=0.077). Planned comparisons at longer-term follow-up indicated that the omega-3 group had significantly lower scores than the placebo group on all PANSS measures (<0.05). The interaction between medication group and occasion was not significant for the MADRS, but the omega-3 group had significantly lower scores than the placebo group at longer-term follow-up (*P*=0.021). Table 1 shows changes in symptoms and functioning from baseline to longer-term follow-up.

### Prescription of antipsychotic medication

The prescription of antipsychotic medication by an independent medical practitioner can be assumed to represent the severity of psychotic phenomena in the sample. Therefore, we investigated whether omega-3 PUFA supplementation reduced the proportion of individuals who needed to be prescribed antipsychotic medication by independent medical practitioners during the entire follow-up period. The proportion of individuals who reported having been prescribed antipsychotic medication at follow-up was 29.4% (10/34) in the omega-3 PUFA group and 54.3% (19/35) in the placebo group. Pearson's *χ*^2^-test indicated a significant group difference (*χ*^2^=4.4, df=1, *P*=0.04).

### Other psychiatric disorders

Diagnostic outcomes at longer-term follow-up are displayed in Table 2. Of the entire sample, 68.1% (47/69) met criteria for at least one disorder during the follow-up period. Of the participants in the placebo group, 82.9% (29/35) met criteria for at least one DSM-IV Axis I disorder during the follow-up period compared with 52.9% (18/34) of the participants in the omega-3 group (Pearson's *χ*^2^-test: *χ*^2^=7.1, df=1, *P*=0.008).

### Outcomes in non-transitioned cases

Since other studies in ultrahigh risk for psychosis samples have reported poor functioning in non-transitioned cases^19^, with some individuals having ongoing attenuated psychotic symptoms and/or non-psychotic disorders^20^, we further examined the longer-term outcomes in those subjects who received omega-3 PUFAs or placebo and did not convert to psychosis. It should be noted that the omega-3 group still comprised almost the complete sample of enrolled subjects, presumably retaining cases more susceptible for mental disorders, while in the placebo group a larger proportion had converted to psychotic disorder. Table 3 shows the frequencies of attenuated psychosis status, functioning levels, employment, non-psychotic psychiatric disorders and treatment history at follow-up in the non-transitioned individuals, by intervention group. In the omega-3 group, attenuated psychosis was present in approximately a quarter of individuals but only 6.7% (2/30) had severe functional impairment and 70.0% (21/30) were employed full-time. Furthermore, less than half of individuals who had received omega-3 PUFAs met criteria for any Axis I disorder at follow-up. These frequencies indicate a high degree of symptomatic remission and functional recovery in the omega-3 PUFA group. Two individuals from the omega-3 PUFA group reported taking fish oil capsules for longer than 1 month during the follow-up period.

The co-occurrence of attenuated psychotic symptoms and functional impairment is presented in Table 4. One individual from the omega-3 group was still experiencing attenuated psychotic symptoms and had severe functional impairment (indicated by a GAF score ≤50) at the longer-term follow-up. The majority of individuals from both treatment groups, including those with attenuated psychotic symptoms, had either mild or moderate functional impairment or good functioning at follow-up.

## Discussion

This is the first study to show, to the best of our knowledge, that a 12-week intervention with omega-3 PUFAs prevented transition to full-threshold psychotic disorder and led to sustained symptomatic and functional improvements in young people with an at-risk mental state for 7 years (median). These findings are supported by the significantly reduced rate of prescription of antipsychotic medication in the omega-3 PUFA group during the entire follow-up period. The overall psychiatric morbidity, indicated by meeting criteria for at least one DSM-IV Axis I disorder during the follow-up period, was also significantly lower in the omega-3 PUFA group. The majority of the individuals from the omega-3 group did not show severe functional impairment, were employed full-time, and no longer experienced attenuated psychotic symptoms at follow-up. The results in the non-transitioned group emphasize the longer-term preventive and therapeutic effect in some of the participants who received omega-3 PUFAs.

Ultrahigh risk for psychosis patients are at longer-term risk for psychotic disorder^8^, and non-psychotic disorders are very common in those individuals who do not transition^20^. In this study, the rate of transition to psychosis in the placebo group was 40%. This rate is consistent with the largest follow-up study of ultrahigh risk patients reported to date, in which the psychosis rate was 35% over a 10-year period^8^. In the only published longer-term follow-up study of non-transitioned cases in young people at ultrahigh risk for psychosis, 68.1% met criteria for at least one disorder during the follow-up period^20^. This rate is remarkably similar to the rate observed in non-transitioned participants who received placebo in our study (68.4%). The difference between the treatment groups in the rates of meeting criteria for an Axis I disorder in non-transitioned subjects was 21.7% (see Table 3), while the difference between the treatment groups in the cumulative risk of progression to full-threshold psychosis was 30.6%. The finding that supplementation with omega-3 PUFAs prevented the onset of psychotic disorder and reduced rates of non-psychotic Axis I disorders offers hope there may be alternatives to psychopharmacological treatment as early interventions in young people at risk for psychosis and raises questions regarding the potential mechanisms of action explaining these effects.

Neuronal circuits in the brain are shaped during critical periods of development^21^. It is therefore possible that an effective intervention during a circumscribed period of increased susceptibility can have longer-term effects. Omega-3 PUFAs provide a range of neurochemical activities via modulation of neurotransmitter (noradrenaline, dopamine and serotonin) reuptake, degradation, synthesis and receptor binding, as well as anti-inflammatory and anti-apoptotic effects, and the enhancement of cell membrane fluidity and neurogenesis^22^. While the mechanisms of action in the present trial remains unclear, the findings imply that omega-3 PUFAs may have stopped processes associated with the manifestation of psychotic disorders.

Two recent animal studies provide preliminary evidence supporting the longer-term efficacy of brief neuroprotective treatments. The first study used a developmental model to test the efficacy of the antioxidant *N*-acetyl cysteine against oxidative stress in rats with a neonatal ventral hippocampal lesion. This study showed that *N*-acetyl treatment during adolescence prevented adult brain structural deficits, as well as electrophysiological and behavioural deficits relevant to schizophrenia in this model^23^. Adolescence may therefore be a critical developmental stage in which pathophysiological conditions (for example, oxidative stress) can affect the developing brain, but at the same time it may also provide a window of opportunity for preventive intervention. Our study findings are consistent with the view that intervention during a critical period may change the developmental trajectory, ultimately leading to a different outcome in adulthood.

More relevant to the omega-3 PUFAs, a second study investigated behavioural measures and assayed markers of dopamine-related neurotransmission in adolescent and adult rats^24^. Interestingly, dietary omega-3 PUFA deficiency produced behavioural deficiencies and alterations in brain markers of dopamine-related neurotransmission that were distinct in adolescents compared with the adults in this study. While adolescent rats displayed significantly enhanced dopamine availability, in adult rats the changes were in the opposite direction, indicating that the dopamine system was differentially disrupted by omega-3 PUFA deficiency in these age groups. Adolescent rats with an omega-3 PUFA-deficient diet had high levels of tyrosine hydroxylase, the enzyme responsible for catalysing the synthesis of dopamine in the dorsal striatum, essentially resembling the pattern of high dopamine activity. Individuals at ultrahigh risk for psychosis have high dorsal striatal dopamine levels, which correlate positively with transition to psychotic disorders^25^. Omega-3 PUFAs may therefore reduce conversion in subjects at risk for psychosis by preventing the pathophysiological changes associated with the increase in striatal dopamine. This finding also implies that omega-3 PUFA supplementation may be specifically effective during adolescence. The fact that the majority of participants in our study were in their adolescence, when neurodevelopment in brain regions relevant to schizophrenia occurs (that is, prefrontal cortex and striatum), could therefore be crucial to the observed effect.

Claims of preventing major health problems invite skepticism and create controversy^26^. However, strengths of this study include the double-blind randomized, placebo-controlled design, the use of standardized inclusion and exit criteria, inter-rater reliability testing, the application of an objective measure for treatment adherence (that is, erythrocyte membrane PUFAs were measured pre- and post-intervention^11^) and confirmation by means of the SCID-I/P and/or meticulous case review that all people who met exit criteria had made transitions to genuine psychotic disorders. The context of people being referred to a specialized psychosis detection unit, the age and specific risk criteria, and the relatively modest sample size that does not allow further subgroup analyses are important limitations of this study. Furthermore, in two non-transitioned individuals from the omega-3 PUFA group who took fish oil capsules for longer periods after their last follow-up, this supplementation may have contributed to their outcomes. In conclusion, this first of its kind trial suggests that omega-3 PUFAs may offer a viable longer-term prevention strategy with minimal associated risk in young people at ultrahigh risk of psychosis.

## Methods

### Study design

A randomized, double-blind, placebo-controlled trial consisting of: (1) a 12-week intervention period with 1.2 g per day omega-3 PUFAs or placebo; (2) a 40-week period during which all participants received state-of-the-art clinical care; and (3) a longer-term follow-up assessment. The study was approved by the Medical University of Vienna ethics committee, and written informed consent was obtained from all participants (parental or guardian consent was obtained for those aged <18 years). Longer-term follow-up data were collected between March 2012 and December 2013. All study participants and raters were blind to group allocation (that is, omega-3 PUFAs; placebo).

### Longer-term follow-up procedure

The study was conducted at the Department of Child and Adolescent Psychiatry, Medical University Vienna, Austria. Informed consent for the longer-term follow-up was gained at time of the baseline assessment. We maximized the follow-up rate using a six-step sequential algorithm for follow-up: (1) research files; (2) psychiatric medical records; (3) a Vienna-wide electronic register of health service utilization; (4) telephone directory; (5) national register of residents; and (6) National Death Index. The research files contain the past assessments and contact details, including those of next of kin. The electronic register of health service utilization was used to obtain information regarding whether participants had been registered with the public mental health system since the last follow-up. Resident registration is compulsory in Austria. The register of residents is a government database containing information on the current residence of persons. The telephone directory was searched for the names of participants and any family members whose names were known to obtain contact information. The National Death Index was used to check whether participants were deceased. Once a possible location for an individual had been determined, he or she was sent a standard letter explaining the purpose of the project and advising that a member of the research team would be in contact. Once an individual was contacted, he or she was invited to participate in a follow-up interview.

### Patient eligibility criteria and exclusion criteria

Individuals were eligible for participation if they were aged 13–25 years and met criteria for at least one of three operationally defined groups of specific state and/or trait risk factors for psychosis. The three groups were as follows: (1) attenuated positive psychotic symptoms; (2) brief, limited intermittent psychotic symptoms (transient psychosis); and (3) trait plus state risk factors (that is, genetic risk plus a decrease in functioning). The rationale and validation for these ultrahigh risk groups has been previously described^12^,^27^,^28^. Following Morrison *et al*.^17^, we used the PANSS^13^ to operationalize the first two groups by applying the following cutoff scores: attenuated psychotic symptoms were defined by the presence of symptom scores of 3 on the delusions scale, 2–3 on the hallucinations scale, 3–4 on suspiciousness or 3–4 on the conceptual disorganization scale (frequency of symptoms several times per week for a period of at least a week and not longer than 5 years, and have occurred within the last year); transient psychosis was defined by the presence of symptom scores of 4 or more on the hallucinations scale, 4 or more on the delusions scale, or 5 or more on the conceptual disorganization scale (symptoms not sustained beyond a week, resolve without antipsychotic medication and have occurred within the last year). The third group, comprising trait plus state risk factors, was defined as having a schizotypal personality disorder (as defined by DSM-IV) or a first-degree relative with a DSM-IV psychotic disorder, and a significant decrease in functioning resulting in a decrease of 30% on the GAF^15^ from the premorbid level, maintained for at least a month and not longer than 5 years. The decrease in functioning needed to have occurred within the previous year.

Individuals were excluded from the study if they had the following: (1) a history of a previous psychotic episode (treated or untreated) or substance-induced psychotic disorder at index presentation; (2) acute suicidal or aggressive behaviour (PANSS hostility or suicidality=7); (3) drug abuse that contributed decisively to the presentation of the index episode (dependency on morphine, cocaine, amphetamine, but not cannabis); (4) alcohol abuse if considered as a major problem; (5) epilepsy; (6) IQ<70; (7) structural changes in magnetic resonance imaging or computed tomography scan (for example, tumours), except for enlargement of ventricles or sulci; (8) previous treatment with an antipsychotic or mood stabilizing agent (more than three daily doses); (9) laboratory values >10% outside the normal range for transaminases, CRP or bleeding parameters; (10) organic brain syndrome; (11) had taken or were taking omega-3 supplements currently or within 8 weeks of being included in the trial; and (12) another severe, intercurrent illness that in the opinion of the investigator may put them at risk or influence the results of the trial, or affect ability to take part in the trial. More information on trial design, interventions, randomization, blinding and study measures is provided by Amminger *et al*.^11^.

### Outcome measures

The primary efficacy measure for the treatment comparison was the rate of conversion to psychosis, which was operationally defined based on criteria by Yung *et al*.^12^, using cutoff points on the PANSS) (4 or more on hallucinations, 4 or more on delusions and 5 or more on conceptual disorganization), the frequency of symptoms (at least several times a week), and their duration (more than 1 week). The exit criteria marked the threshold (linked to positive symptoms) at which treatment with antipsychotic medication is usually initiated^18^. Secondary efficacy measures included the PANSS^13^, the MADRS^14^ and the GAF^15^. The SCID-I/P^16^ was used to ascertain psychiatric diagnoses at baseline, 12-month, and longer-term follow-up. The SCID ratings at 12-month and longer-term follow-up were supplemented by additional sources, including a medical records review and an informant interview, usually conducted with a parent or caregiver.

### Inter-rater reliability

Raters were experienced clinicians who were thoroughly trained in the administration of outcome measures before the beginning of the study. Inter-rater reliability estimates for PANSS subscales, MADRS and GAF were excellent (all intraclass correlation coefficients ⩾0.92)^11^. To maintain reliability between raters, videotaped interviews were used approximately every 3 months across the initial 12 months of the study and before the longer-term follow-up was commenced.

### Data analysis

Analyses were performed on an intention-to-treat basis. Kaplan–Meier survival analysis assessed differences in time to transition to psychosis between the treatment arms at longer-term follow-up using the log-rank test. A secondary sensitivity analysis was performed under the assumption that all participants who were lost to follow-up prior to the longer-term follow-up assessment had converted to psychosis. For secondary outcome measures, analyses were carried using the mixed model repeated-measures analysis of variance. The within-groups factor was measurement occasion, and medication group served as the between-groups factor. A Toeplitz co-variance structure was used to model relations between observations on different occasions. A series of planned comparisons contrasted change from baseline to the 12-week, 6-month, 12-month and longer-term follow-up assessments between omega-3 and placebo. Mixed model repeated-measures analysis of variance differs from traditional repeated-measures analysis of variance in that all available data are included in the model and the associations between the different times are also modelled. Analyses were undertaken using the MIXED procedure in SPSS, version 21. In accordance with our original analysis, the score at the time of transition to psychosis (that is, the time when a participant exited the trial and commenced antipsychotic medication) was used in the analysis for those individuals who converted to psychosis. A transition score has been prospectively assigned to individuals in the original study who developed psychosis before the 12-month follow-up^11^. In those individuals who made a transition to psychosis after the 12-month follow-up, transition scores were estimated retrospectively at longer-term follow-up based on interview and/or hospital record information. For comparisons of categorical variables, we calculated Pearson's *χ*^2^-tests. A significance level of 0.05 was used for all statistical tests, and all tests were two tailed.

## Additional information

**How to cite this article:** Amminger, G. P. *et al*. Longer-term outcome in the prevention of psychotic disorders by the Vienna omega-3 study. *Nat. Commun.* 6:7934 doi: 10.1038/ncomms8934 (2015).

## Figures and Tables

**Table 1:** Changes between baseline and longer-term follow-up for secondary outcome measures.

	**Baseline**		**Change from baseline**		*P-**value**
	**Omega-3 (*****n*****=41)**	**Placebo (*****n*****=40)**	**Omega-3 (*****n*****=41)**	**Placebo (*****n*****=40)**	
*PANSS score*					
Total	59.9 (2.8)	57.2 (2.8)	−13.9 (3.3)	0.2 (3.3)	0.003
Positive	15.0 (0.7)	14.2 (0.7)	−5.1 (0.9)	−0.8 (0.9)	0.002
Negative	14.0 (0.9)	13.6 (0.9)	−3.1 (1.1)	0.4 (1.1)	0.024
General	30.9 (1.4)	29.4 (1.4)	−5.6 (1.8)	0.6 (1.8)	0.015
MADRS score	17.6 (1.5)	18.8 (1.6)	−7.3 (2.0)	−2.7 (2.0)	0.117
GAF score	61.0 (2.5)	60.0 (2.5)	7.7 (2.7)	−0.8 (2.7)	0.028

GAF, Global Assessment of Functioning; MADRS, Montgomery–Asberg Depression Rating Scale; PANSS, Positive and Negative Syndrome Scale.

NB: values that refer to mean (s.e.).

^*^P-values that refer to contrasts from the repeated-measures mixed model.

**Table 2:** Rates of Axis I diagnoses during follow-up in young people at ultrahigh risk for psychosis at baseline.

**Diagnosis**	**Entire sample (*****N*****=69)**		**Omega-3 group (*****N*****=34)**		**Placebo group (*****N*****=35)**	
	***N***	**%**	***N***	**%**	***N***	**%**
Any disorder	47	68.1	18	52.9	29	82.9
						
Psychotic disorder	20	29.0	4	11.8	16	45.7
Schizophrenia, paranoid type	11	15.9	2	5.9	9	25.7
Schizophreniform disorder	2	2.9	1	2.9	1	2.9
Schizoaffective disorder	4	5.8	1	2.9	3	8.6
Psychosis NOS	2	2.9	0	0.0	2	5.7
Bipolar I disorder with psychotic features	1	1.4	0	0.0	1	2.9
						
Mood disorder	26	37.7	13	38.2	13	37.1
Major depressive disorder	24	34.8	12	35.3	12	34.3
Bipolar II disorder	2	2.9	1	2.9	1	2.9
						
Anxiety disorder	19	27.5	9	26.5	10	28.6
Anxiety disorder with agoraphobia	2	2.9	1	2.9	1	2.9
Anxiety disorder without agoraphobia	4	5.8	1	2.9	3	8.6
Anxiety disorder NOS	4	5.8	2	5.9	2	5.7
Social Phobia	5	7.2	3	8.8	2	5.7
Obsessive compulsive disorder	4	5.8	2	5.9	2	5.7
						
Substance use disorders	7	10.1	2	5.9	5	14.3
Alcohol abuse	2	2.9	1	2.9	1	2.9
Alcohol dependence	1	1.4	1	2.9	0	0.0
Cannabis abuse	4	5.8	0	0.0	4	11.4
						
Other disorders	3	4.3	0	0.0	3	8.6
Bulimia nervosa	1	1.4	0	0.0	1	2.9
Eating disorder NOS	1	1.4	0	0.0	1	2.9
Somatization disorder	1	1.4	0	0.0	1	2.9

**Table 3:** Longer-term outcomes in non-transitioned cases in a sample of young people at ultrahigh risk for psychosis.

**Outcome**	**All Non-transitioned (*****N*****=49)**		**Omega-3 group (*****N*****=30)**		**Placebo group (*****N*****=19)**	
	***N***	**%**	***N***	**%**	***N***	**%**
Attenuated psychosis	11	22.4	8	26.7	3	15.8
						
Functioning*						
Good functioning	24	49.0	14	46.7	10	52.6
Mild or moderate impairment	21	42.4	14	46.7	7	36.8
Severe impairment	4	8.2	2	6.7	2	10.5
						
*Employment*						
Full-time	33	67.3	21	70.0	12	63.2
Part-time	3	6.1	1	3.3	2	10.5
Unemployed	13	26.5	8	26.7	5	26.3
						
*Non-psychotic disorder*						
Any disorder	27	55.1	14	46.7	13	68.4
Mood disorder	18	36.7	12	40.0	6	31.6
Anxiety disorder	13	26.5	8	26.7	5	26.3
Substance use disorders	5	10.2	2	6.7	3	15.8
Other disorder†	1	2.0	0	0.0	1	5.3
						
*Psychiatric treatment*‡						
Outpatient psychiatric care	30	61.2	17	56.7	13	68.4
Inpatient psychiatric care	10	20.4	5	16.7	5	16.7
Supplementation§	2	4.1	2	6.7	0	0.0

^*^Good functioning GAF score >70; Mild or moderate impairment GAF score between 51 and 70; severe impairment GAF score ≤50.

^†^Somatization disorder.

^‡^Service use during the follow-up period.

^§^Supplementation with omega-3 PUFAs >1 month.

**Table 4:** Co-occurrence of functioning impairment and presence or absence of attenuated psychotic symptoms at follow-up by intervention group.

Functioning*	**Presence or absence of attenuated psychotic symptoms**											
	**All non-transitioned**				**Omega-3 group**				**Placebo group**			
	**Present (*****N*****=11)**		**Absent (*****N*****=38)**		**Present (*****N*****=8)**		**Absent (*****N*****=22)**		**Present (*****N*****=3)**		**Absent (*****N*****=16)**	
	***N***	**%**	***N***	**%**	***N***	**%**	***N***	**%**	***N***	**%**	***N***	**%**
Good functioning	4	36.4	20	52.6	3	37.5	11	50.0	1	33.3	9	56.3
Mild or moderate impairment	6	54.5	15	39.5	4	50.0	10	45.5	2	66.7	5	31.3
Severe impairment	1	9.1	3	7.9	1	12.5	1	4.5	0	0.0	2	12.5

^*^Good functioning GAF score >70; mild or moderate impairment GAF score between 51 and 70; severe impairment GAF score ≤50.
